# Matrix symmetry and normality in multimode fiber transport

**DOI:** 10.1063/5.0337399

**Published:** 2026-09-01

**Authors:** Shreyas Bharadwaj, Gyeong Hun Kim, Brett E. Bouma, Martin Villiger

**Affiliations:** 1 Department of Electrical Engineering and Computer Science, Massachusetts Institute of Technology, Cambridge, Massachusetts 02139, USA; 2 Wellman Center for Photomedicine, Harvard Medical School and Massachusetts General Hospital, Boston, Massachusetts 02114, USA; 3 Institute for Medical Engineering and Science, Massachusetts Institute of Technology, Cambridge, Massachusetts 02142, USA

## Abstract

Multimode fibers (MMFs) support high-dimensional optical transport within a compact form factor, with growing applications in biomedical imaging, telecommunications, and quantum engineering. Realizing this potential requires accurate knowledge of the fiber transmission matrix (TM); yet experimentally measured TMs are often corrupted by system-level distortions that obscure the underlying modal physics and limit wavefront control. Here, we show that the reciprocity of optical waves imposes symmetry constraints that provide self-consistency conditions for identifying and correcting such distortions. Enforcing these constraints enables improved recovery of the transported fields and allows the computational synthesis of confocal images from reflection measurements through the fiber. We further introduce matrix normality as a physically meaningful metric that quantifies the orthogonality of the propagating modes encoded in the TM. Using controlled static compression of the fiber, we demonstrate experimentally that departures from normality at a single wavelength predict a monotonic reduction in the spectral bandwidth of the principal mode basis. These results reveal a direct connection between spatial modal structure and spectral stability in MMF transport. More broadly, they establish a unified framework for understanding and controlling broadband light propagation in MMFs and complex waveguides.

## I. INTRODUCTION

Owing to their thin form-factor, support of high-dimensional optical transport, and scalable manufacturing, multimode fibers (MMFs) are an attractive platform for coherent applications in photonics. Despite well-established theoretical models of propagation in an ideal fiber via orthogonal, propagation-invariant eigenmodes,^1^ real-world MMFs exhibit imperfections that induce modal coupling, significantly changing their transmission characteristics. This coupling scrambles the spatial and polarization modes, transforming the fiber into a complex, quasi-random medium.^2,3^ In such systems, the experimentally measured transmission matrix^4^ (TM) is the essential descriptor that encapsulates all such effects. Exploiting the TM has enabled MMF-based lensless imaging,^5–8^ micromanipulation,^9^ space-division multiplexing,^10^ and quantum networks.^11^ The TM has also served as the primary tool to probe the fundamental physics of such complex waveguides, for instance, by characterizing their response to physical deformation^2,12^ or dispersion.^13^

However, the reliability of these matrix-based approaches is limited by the fidelity of the experimental measurement of the fiber TM. In high-dimensional systems, system-level aberrations, such as misalignments, wavefront curvature, or polarization crosstalk, become indistinguishable from the intrinsic modal mixing of the fiber itself. This ambiguity precludes the accurate recovery of the transported fields and obscures the underlying wave physics.

To resolve this, fundamental physical symmetries, specifically reciprocity, can be exploited as self-consistency conditions to constrain the measured matrices. Reciprocity dictates the transpose-symmetry of the linear scattering operator and enforces corresponding relationships on its transmission and reflection sub-components.^14–16^ In practice, experimentally measured matrices often fail to exhibit these symmetries. This departure usually arises from asymmetries in the experimental apparatus, rather than truly non-reciprocal optical effects of a magneto-optic, nonlinear, or active nature. Based on this concept, we previously illustrated the restoration of reciprocity-imposed symmetries in scalar MMF forward and round-trip TMs by computational application of corrective wavefronts based on the Zernike mode basis.^17^ The present investigation builds on this idea by employing reciprocity-imposed symmetries themselves as self-consistency conditions to guide the compensation of experimental aberrations in polarization-resolved TMs of MMFs. An optimization procedure is developed that enforces these symmetries computationally, without recourse to heuristic parameterizations of the underlying aberrations. The corrections obtained through this approach are found to correspond to familiar experimental effects, indicating that such modes arise as violations of the underlying symmetry constraints. The restoration of these symmetries is furthermore found to significantly impact the fidelity of confocal gating when imaging through the MMF in reflection.

Once system-induced asymmetries are removed, the corrected TM becomes suitable for a physically meaningful modal analysis. On the basis of the physically faithful fiber TMs, we employ matrix normality to characterize the orthogonality of the propagating modes. Normal matrices admit unitary diagonalization and, therefore, describe transmission in terms of orthonormal channels. The degree of normality is quantified and studied through eigenspectral analysis of the MMF TMs. Matrix normality is then shown to be a critical factor governing the spectral stability of a system. This stability, in turn, defines its response to perturbative changes and is investigated in MMF through examination of the spectral correlation bandwidth of its principal mode basis.

## II. RESTORATION OF RECIPROCITY-IMPOSED SYMMETRIES

The scattering matrix **S** of an *n*-port system is defined as

$$S=\left[\begin{array}{cc} R_{ll} & T_{rl}\\ T_{lr} & R_{rr} \end{array}\right],$$ (1)

where **R** and **T** denote reflection (RM) and transmission (TM) matrices, respectively, with respect to the flux-carrying modes on the left (*l*) and right (*r*) of the medium.^14^ The system’s response to a given input field

$$x=\left[\begin{array}{c} x_{l}\\ x_{r} \end{array}\right],$$ (2)

is **y** = **S** · **x**. The scattered fields on the left and right sides are then

$$y_{l}=R_{ll}\cdot x_{l}+T_{rl}\cdot x_{r},$$ (3)

and

$$y_{r}=T_{lr}\cdot x_{l}+R_{rr}\cdot x_{r}.$$ (4)

In reciprocal media, **S** is transpose-symmetric, i.e., it satisfies^14^ **S** = **S**^*T*^. In wave-guiding media like MMF with minimal same-side reflections, the full scattering matrix may be approximated by only its transmission components in the forward (**T**_fw_) and backward (**T**_bw_) directions. In such systems, the measured reflection matrix from a given facet is a result of (1) propagation through the fiber (**T**_fw_ or **T**_bw_), (2) Fresnel reflection at the opposite fiber facet (**I**), and retro-propagation along the reverse direction (**T**_bw_ or **T**_fw_, respectively). The full scattering matrix becomes

$$S_{\text{MMF}}\approx \left[\begin{array}{cc} T_{\text{bw}}\cdot T_{\text{fw}} & T_{\text{bw}}\\ T_{fw} & T_{\text{fw}}\cdot T_{\text{bw}} \end{array}\right],$$ (5)

where $S_{\text{MMF}}=S_{\text{MMF}}^{T}$ imposes $T_{\text{bw}}=T_{\text{fw}}^{T}$.

In any real optical system, however, aberrations in the illumination and detection paths break these ideal symmetries. This apparent departure from reciprocity provides a physical basis for correcting system-level distortions.

To develop and test this principle, the TMs of MMF were measured. The matrices were constructed by scanning a square grid of focused input spots across the fiber facet from a fiber-coupled, wavelength-tunable semiconductor laser operating at 1310 nm in a Mach–Zehnder interferometric configuration. The polarization of each input mode was switched between orthogonal linear states (horizontal, denoted H, and vertical, denoted V) for each scan position. On the detection side, the transmitted or reflected output from the sample was combined with the reference wave in an off-axis configuration and imaged onto an InGaAs camera through a beam displacer, which enabled polarization-resolved detection. A full measurement took about five seconds, and the matrices were expressed on the spot-to-camera pixel basis in block-Jones form

$$M=\left[\begin{array}{ccc} M_{\text{HH}} & & M_{\text{HV}}\\ M_{\text{VH}} & & M_{\text{VV}} \end{array}\right],$$ (6)

where the indices of each sub-block correspond to the detected and illuminated polarization states, in that order. Full experimental details are provided in Appendix A.

Using this procedure, the forward TM of a 1-m-long step-index MMF (0.22 NA, 50 *μ*m diameter, supporting ∼170 modes per polarization), **T**_fw_, was obtained. By using the distal facet of the MMF as a reflector, the double-pass MMF TM, **T**_2×_, was also measured using a similar procedure. Reciprocity imposes two fundamental constraints on **T**_2×_: (1) as a RM, it must be transpose-symmetric $\left(T_{2\times }=T_{2\times }^{T}\right)$ and (2) it must be equal to the transpose-product of the forward TMs^14,17^ $\left(T_{2\times }=T_{\text{bw}}\cdot T_{\text{fw}}=T_{\text{fw}}^{T}\cdot T_{\text{fw}}\right)$.

### A. Experimental basis registration

As measured, the TMs were expressed on a spot-to-camera pixel basis, corresponding to the illumination and detection modes in our experiment. However, owing to the difference between the two, an explicit co-registration was conducted to enable a meaningful analysis of reciprocity. The output fields, expressed originally in a large grid of camera pixels, were downsampled in the spatial frequency domain to match the dimensionality of the input spot grid. Due to the limited numerical aperture of the MMF, this subsampling retained all detected spatial frequency components of the output field and resulted in square TMs expressed in the spot-to-subsampled pixel basis. To register the bases, a spot-to-subsampled pixel transformation operator, denoted **W**, was constructed by experimentally measuring the full camera image of each spot and subsampling it in the spatial frequency domain to match the dimensions of the spot grid. Applying the inverse of this matrix (equivalently, its transpose) to the input side resulted in TMs whose input and output bases were expressed as subsampled camera pixels. This transformation resulted in square matrices with matching input and output bases. A more detailed procedure for constructing **W** and registering the TMs is described in Appendix B. Basis registration was an essential prerequisite, as any geometric mismatch between the illumination and detection bases obscures operator-level properties of the measured TM. Notably, the explicit alignment of input and output bases is unnecessary in some wave-based systems, such as when using identical ultrasound transducer arrays for excitation and detection, for example;^18^ however, the illumination and detection of light waves occur with distinct apparatus and, therefore, necessitate this procedure.

### B. Symmetrization of MMF round-trip TM

Though corrected for mismatched input and output bases, the measured matrices nevertheless departed from the symmetries imposed by reciprocity: **T**_2×_ was not transpose-symmetric, and $T_{\text{fw}}^{T}\cdot T_{\text{fw}}≉T_{2\times }$. Hence, our objective was, therefore, to computationally restore the expected symmetries and thereby correct for experimental aberrations. The correction of **T**_2×_ was first considered, employing its intrinsic transpose-symmetry as a measure of fidelity.

To this end, **T**_2×_ was modeled as a composite operator resulting from linear transformations attributable to both the fiber $\left(T_{2\times }^{\prime }\right)$ and the experimental apparatus,

$$T_{2\times }=B\cdot T_{2\times }^{\prime }\cdot A,$$ (7)

where $T_{2\times }^{\prime }=T_{2\times }^{\prime T}$ is the true round-trip matrix and **A** and **B** encapsulate the effects of the illumination and detection optics, respectively.

The restoration of transpose-symmetry to **T**_2×_ corresponds to correcting the asymmetry between the illumination and detection paths. However, the particular choice of matching the former to the latter, or vice versa, is inherently ambiguous. This can be seen mathematically: both (1) left-multiplication of **T**_2×_ by **A**^*T*^ ⋅**B**^−1^ (output-side) and (2) right-multiplication by **A**^−1^ ⋅**B**^*T*^ (input-side) result in symmetric matrices ($A^{T}\cdot T_{2\times }^{\prime }\cdot A$ and $B\cdot T_{2\times }^{\prime }\cdot B^{T}$, respectively).

However, our illumination consisted only of a scanned, collimated beam transmitted through a beam splitter and focused by an objective lens. On the other hand, our detection path imaging system was a telescope in a 4*f* configuration with a simple achromat employed as a tube lens, one or more mirrors, and a beam displacer. Therefore, the detection-side optics were presumed to be the dominant source of experimental aberrations (see Appendix A for experimental details). Accordingly, we modeled the correction term as a left-multiplicative linear operator, denoted **X**_2×_; however, a right-multiplicative formulation would be fundamentally equivalent from the perspective of reciprocity. To determine **X**_2×_, an optimization problem was formulated to penalize deviation from the underlying symmetry constraint,

$${\hat{X}}_{2\times }={\mathrm{arg min}}_{X_{2\times }}\|{\tilde{T}}_{2\times }-{\tilde{T}}_{2\times }^{T}{\|}_{F}^{2},$$ (8)

where the corrected operator ${\tilde{T}}_{2\times }=X_{2\times }\cdot T_{2\times }$ and ‖⋅‖_*F*_ denotes the Frobenius norm. **X**_2×_ was iteratively optimized by gradient descent, as explained below and illustrated in Algorithm 1.

**ALGORITHM 1.** Restoring reciprocity-imposed symmetries.

**Input**: Raw round-trip matrix **T**_2×_, basis registration matrix **W**, spatial Fourier transform matrix F, learning rate α
**Output**: Symmetrized matrix ${\hat{T}}_{2\times }$, near-field correction **Ψ**_2×_, far-field correction $\Phi_{2\times }$
1: **Initialize**:
2: Apply **W** to co-register input and output bases of **T**_2×_
3: **Φ**_2×_ ←**I** ⊳ Initialize far-field diagonal correction as identity
4: **Ψ**_HH_, **Ψ**_HV_, **Ψ**_VH_, **Ψ**_VV_ ←**I** ⊳ Initialize near-field diagonal correction in each Jones block as identity
5: L←∞ ⊳ Initialize error
6: ΔL←∞
7: **while** anti-symmetric power ≥2% **and** ΔL≥0.1% **do**
8: **X**_2×_ ←**F**^†^⋅**Φ**_2×_⋅**F** ⋅**Ψ**_2×_ ⊳ Construct correction operator
9: ${\tilde{T}}_{2\times }\leftarrow X_{2\times }\cdot T_{2\times }$ ⊳ Apply left-multiplicative correction
10: $L_{\text{new}}\leftarrow \|{\tilde{T}}_{2\times }-{\tilde{T}}_{2\times }^{T}{\|}_{F}^{2}$ ⊳ Evaluate deviation from transpose-symmetry
11: $\Delta L\leftarrow L-L_{\text{new}}$
12: $L\leftarrow L_{\text{new}}$
13: $K\leftarrow {\tilde{T}}_{2\times }-{\tilde{T}}_{2\times }^{T}$ ⊳ Compute anti-symmetric residual
14: $\nabla_{\Phi_{2\times }}L\leftarrow \mathrm{diag}\left(F^{†}\cdot K\cdot T_{2\times }^{†}\cdot \Psi_{2\times }^{†}\cdot F\right)$ ⊳ Compute far-field gradient and enforce diagonality	
15: $\nabla_{\Psi_{2\times }}L\leftarrow {\mathrm{diag}}_{b}\left(F\cdot \Phi_{2\times }^{†}\cdot F^{†}\cdot K\cdot T_{2\times }^{†}\right)$ ⊳ Compute near-field gradient and enforce diagonality in each Jones block	
16: $\Psi_{2\times }\leftarrow \Psi_{2\times }-\alpha \cdot \nabla_{\Psi_{2\times }}L$ ⊳ Update near-field correction	
17: $\Phi_{2\times }\leftarrow \Phi_{2\times }-\alpha \cdot \nabla_{\Phi_{2\times }}L$ ⊳ Update far-field correction	
18: **end while**	
19: **return** ${\hat{T}}_{2\times }={\tilde{T}}_{2\times },\Psi_{2\times },\Phi_{2\times }$	

While the initial gradient updates of **X**_2×_ exhibited a predominantly diagonal structure in each polarization sub-block, consistent with physically plausible, spatially local aberrations, subsequent iterations introduced off-diagonal elements that grew in magnitude and lacked spatial structure.

To constrain **X**_2×_, the knowledge of our experimental system’s imaging geometry was incorporated by modeling the asymmetry between illumination and detection paths using diagonal matrices in the near- and far fields, denoted **Ψ**_2×_ and **Φ**_2×_, respectively, reflecting the assumption of spatially local aberrations. **Ψ**_2×_ captured near-field polarization state rotation and aberrations, while **Φ**_2×_ modeled far-field aberrations:

$$\Psi_{2\times }=\left[\begin{array}{cc} \Psi_{\text{HH}} & \Psi_{\text{HV}}\\ \Psi_{\text{VH}} & \Psi_{\text{VV}} \end{array}\right],$$ (9)

$$\Phi_{2\times }=\left[\begin{array}{cc} \Phi_{\text{H}} & 0\\ 0 & \Phi_{\text{V}} \end{array}\right].$$ (10)

Here, **Ψ**_*ij*_ and **Φ**_*k*_ sub-blocks are themselves diagonal matrices. The full correction operator ansatz was built by composing the near- (spatial) and far-field (spatial frequency) operators in their respective domains,

$$X_{2\times }=F^{†}\cdot \Phi_{2\times }\cdot F\cdot \Psi_{2\times },$$ (11)

where **F** and **F**^†^ denote the forward and inverse transformations between the spatial and spatial frequency domains, respectively.

**Ψ**_2×_ and **Φ**_2×_ were optimized until either (i) less than 2% of the total matrix power (i.e., $\|T_{2\times }{\|}_{F}^{2}$) resided in the anti-symmetric component of ${\tilde{T}}_{2\times }$, defined as

$$\frac{{\tilde{T}}_{2\times }-{\tilde{T}}_{2\times }^{T}}{2},$$ (12)

or (ii) the loss function decreased by less than 0.1% between iterations. The final, corrected matrix was denoted ${\hat{T}}_{2\times }={\hat{X}}_{2\times }\cdot T_{2\times }$.

**FIG. 1.**
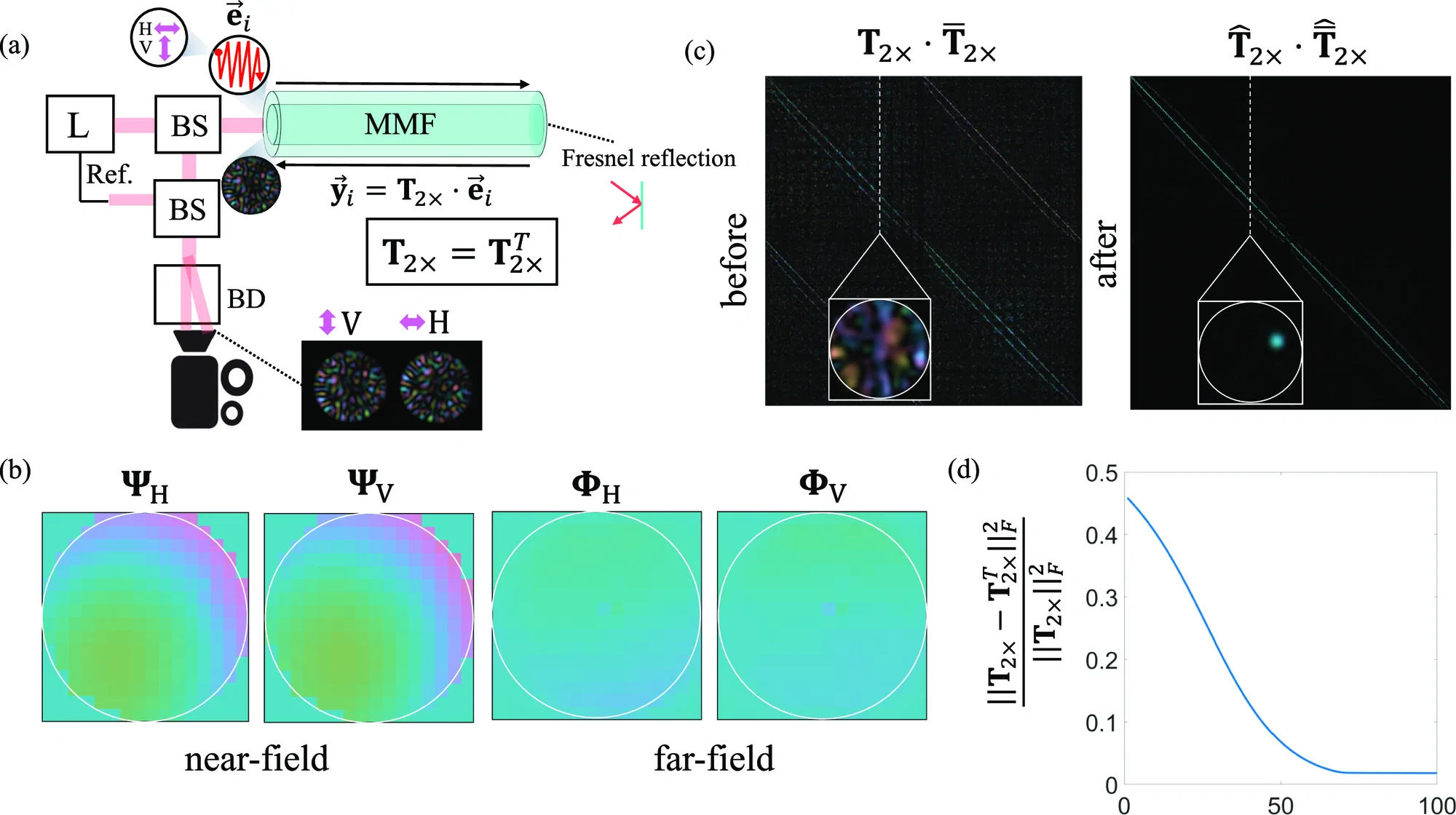
(a) Schematic measurement procedure for the MMF round-trip TM using a spot-grid illumination basis and off-axis holographic detection at the proximal facet (L: laser, BS: beam-splitter, BD: beam-displacer, Ref.: reference arm). (b) Recovered near- and far-field phase and amplitude profiles for both horizontal (H) and vertical (V) polarizations, indicating a primary effect of tilt in the experimental system. The white circular insets show the limited spatial extent of the MMF facet. (c) Time-reversal operator before $\left(T_{2\times }\cdot {\bar{T}}_{2\times }\right)$ and after $\left({\hat{T}}_{2\times }\cdot {\bar{\hat{T}}}_{2\times }\right)$ optimization, illustrating recovery of the identity transformation; (d) Normalized Frobenius norm of the anti-symmetric component vs iteration, showing convergence of the algorithm.

The proposed method was first applied to correct a measured MMF **T**_2×_, whose input and output bases were aligned using the previously described **W** matrix. Following optimization, this quantity was reduced from 38.7% in the uncorrected **T**_2×_ to 1.98% in the corrected matrix, ${\hat{T}}_{2\times }$ [Fig. 1(d)]. Across multiple trials, the procedure consistently reduced the relative fraction of antisymmetric power from 35% to 45% to below 2% within a few seconds of computation time. The largely phase-only nature of the final **Ψ**_2×_ and **Φ**_2×_ emerged naturally from the optimization procedure, without constraining their diagonal elements to be of unit magnitude. The dominant correction, consisting of a linear phase in the near-field component of both polarization terms, indicated a residual tilt in the optical system [Fig. 1(b)]. We observed that the off-diagonal blocks of **Ψ**_2×_ (**Ψ**_HV_, **Ψ**_VH_) were negligible, converging to ≤0.01I after being initialized to **I**.

The physical plausibility of the corrected matrices was evaluated by examining their adherence to Maxwellian time-reversal symmetry. This principle dictates that a scattered field retraces its original path upon complex conjugation of the wavefront and reversal of the propagation direction. In the scattering matrix formalism, time-reversal is described as follows: a field **y**_spot_ resulting from an input **x**_spot_ (i.e., **y**_spot_ = **S** · **x**_spot_) is conjugated and multiplied by the propagation-reversed operator, **S**^*T*^. This process is expected to reconstruct the time-reversed input spot,

$$S^{T}\cdot {\bar{y}}_{\text{spot}}={\bar{x}}_{\text{spot}},$$ (13)

where the overbar denotes element-wise complex conjugation. For a reciprocal medium, the scattering matrix is transpose-symmetric (**S** = **S**^*T*^). The time-reversal condition consequently becomes $S\cdot {\bar{y}}_{\text{spot}}\approx {\bar{x}}_{\text{spot}}$, which provides a direct test of the matrix fidelity. By substitution, this relationship may be expressed as

$$\left(S\cdot \bar{S}\right)\cdot {\bar{x}}_{\text{spot}}\approx {\bar{x}}_{\text{spot}}.$$ (14)

The time-reversal operator is formally defined for the full scattering matrix **S** as the product $S\cdot \bar{S}$, which yields the identity matrix in a lossless, reciprocal system. Because this property applies strictly to the global operator **S**, its individual sub-blocks (the TMs and RMs) are not independently constrained by this condition.

However, the nearly lossless nature of the fiber propagation imposes an analogous constraint on the symmetric round-trip operator **T**_2×_. Therefore, we visualized the product $T_{2\times }\cdot {\bar{T}}_{2\times }$ [Fig. 1(c)] to visually illustrate the effect of the symmetry-restoring procedure. While the power in the anti-symmetric component of the matrix reducing to ≤2% confirms mathematical symmetry, evaluating the time-reversal operator $T_{2\times }\cdot {\bar{T}}_{2\times }$ provides a physically meaningful visual proof that the optimization procedure maintained the ability to reconstruct a spot through phase conjugation.

Relying only on the intrinsic transpose-symmetry of the fiber round-trip matrices, the proposed algorithm was expected to generalize to correcting asymmetries in measurements of the reflection matrices of arbitrary scattering media. Supplementary material File 1 contains the results of additional experiments investigating this concept, which confirmed that our approach was generally applicable and revealed matching system-level aberrations across sample types.

### C. Aligning forward and round-trip TMs

The correction of forward TMs was next considered. Owing to the slight experimental differences in the distal optics between the measurement paths for forward and round-trip TMs (the former using a different objective lens and reflection from a toggled flip mirror), we optimized for an independent correction term **X**_fw_ to compensate for asymmetries in **T**_fw_. The goal was to match the transpose-product $T_{\text{fw}}^{T}\cdot T_{\text{fw}}$ to the corrected ${\hat{T}}_{2\times }$, which serves as a ground-truth under reciprocity. To this end, we applied the same ansatz

$$X_{\text{fw}}=F^{†}\cdot \Phi_{\text{fw}}\cdot F\cdot \Psi_{\text{fw}},$$ (15)

on the left-hand side of the experimentally measured **T**_fw_. Accordingly, an optimization problem was then formulated to penalize deviation from ${\hat{T}}_{2\times }=T_{\text{fw}}^{T}\cdot T_{\text{fw}}$,

$${\hat{X}}_{\text{fw}}={\mathrm{arg min}}_{X_{\text{fw}}}\;\|{\hat{T}}_{2\times }-{\tilde{T}}_{\text{fw}}^{T}\cdot {\tilde{T}}_{\text{fw}}{\|}_{F}^{2},$$ (16)

where the corrected forward TM ${\tilde{T}}_{\text{fw}}=X_{\text{fw}}\cdot T_{\text{fw}}$. As before, **Ψ**_fw_ and **Φ**_fw_ were constrained to diagonal form in the near and far-fields and optimized iteratively using gradient descent. The corrected forward TM is denoted ${\hat{T}}_{\text{fw}}={\hat{X}}_{\text{fw}}\cdot T_{\text{fw}}$.

**FIG. 2.**
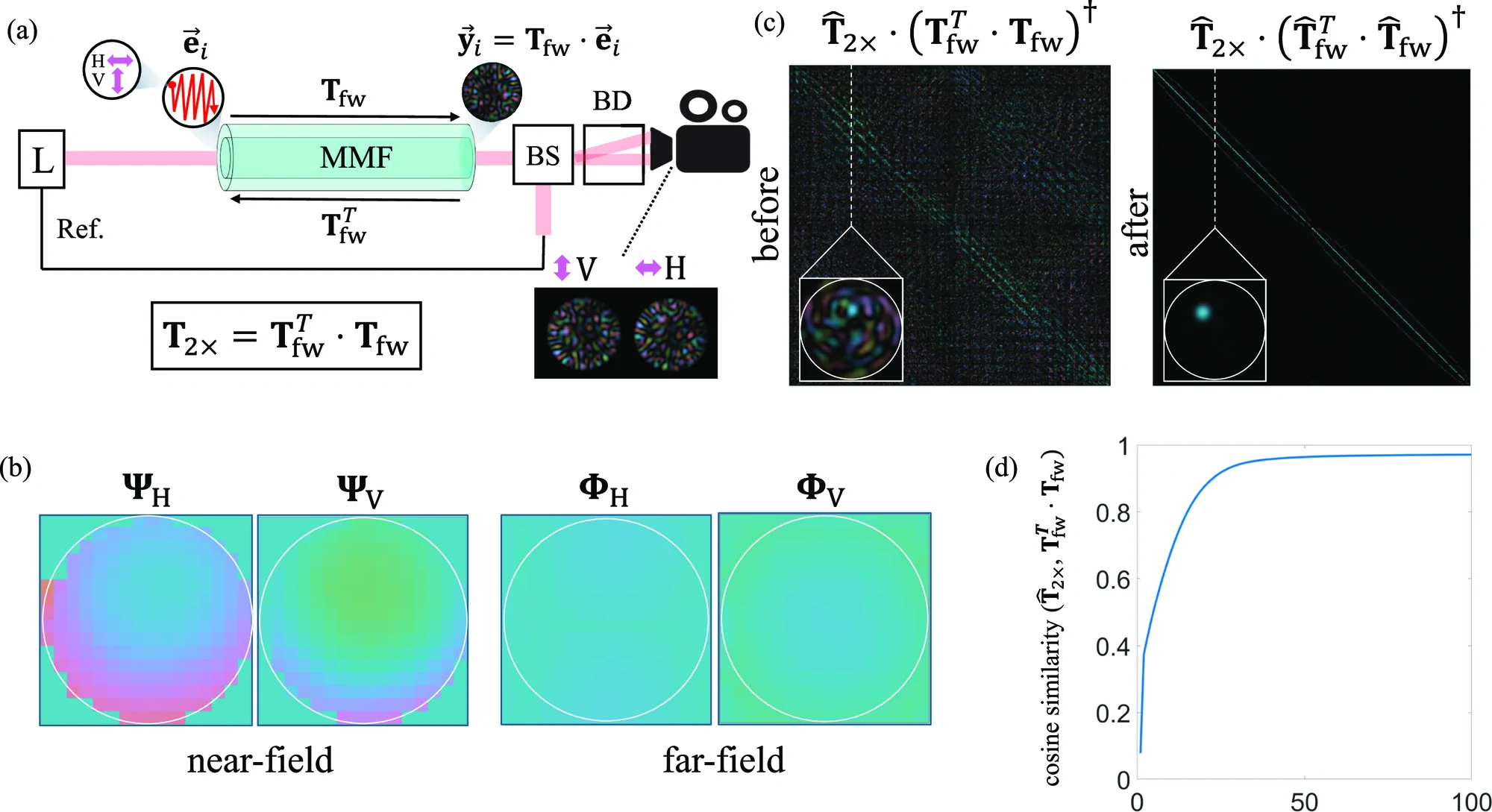
(a) Schematic measurement procedure for the MMF forward TM using a spot-grid illumination basis and off-axis holographic detection at the distal facet (L: laser, BS: beam splitter, BD: beam displacer, Ref.: reference arm). (b) Recovered near- and far-field phase and amplitude profiles for both horizontal (H) and vertical (V) polarizations, indicating a polarization-dependent effect of tilt in the experimental system. (c) Time-reversal operator before $\left({\hat{T}}_{2\times }\cdot {\left(T_{\text{fw}}^{T}\cdot T_{\text{fw}}\right)}^{†}\right)$ and after $\left({\hat{T}}_{2\times }\cdot {\left({\hat{T}}_{\text{fw}}^{T}\cdot {\hat{T}}_{\text{fw}}\right)}^{†}\right)$ optimization, illustrating recovery of the identity transformation. (d) Convergence of the cosine similarity between the evolving transpose-product $T_{\text{fw}}^{T}\cdot T_{\text{fw}}$ and the target round-trip matrix ${\hat{T}}_{2\times }$ to 97.2%.

This procedure improved alignment between single- and double-pass matrices, as shown in Fig. 2. The initial cosine similarity between $T_{\text{fw}}^{T}\cdot T_{\text{fw}}$ and ${\hat{T}}_{2\times }$ was 7.33%, which increased to 97.2% after optimization [Fig. 2(d)]. In a similar fashion to the correction of symmetric operators, time-reversal symmetry was assessed by examining the associated operator ${\hat{T}}_{2\times }\cdot {\left(T_{\text{fw}}^{T}\cdot T_{\text{fw}}\right)}^{†}$. This operator evaluates whether energy sent in a round-trip through the MMF can be returned and refocused by time-reversing the independently measured forward trajectory. Reshaping a single column to the input basis illustrates the recovery of a particular input spot. The proposed algorithm improved the time-reversibility of **T**_fw_, as shown by the reconstruction of an illumination focus in Fig. 2(c). Figure 2(b) illustrates the recovered compensation terms, whose linear, near-field phase corresponds to experimental tilt. One may contrast these phase profiles from those in Fig. 1(b); their structural similarities and differences were attributed to the fact that the distal optics for both forward and round-trip measurements shared a tube lens and beam displacer but differed via distinct objective lenses and an extra mirror in the former.

Numerically, it was observed that the quadratic nature of the correction term $X_{\text{fw}}^{T}\cdot X_{\text{fw}}$ could cause a *π* ambiguity in the recovered phase. Because the recovered compensation terms isolate static system-level aberrations (such as tilt or defocus), their spatial phase profiles are smooth and slowly varying (e.g., corresponding to low-order Zernike polynomials). Consequently, the spatial gradients remain low, typically presenting only a single branch cut in our experiments, thus making standard two-dimensional phase unwrapping algorithms robust in resolving the quadratic *π* ambiguity. The algorithm described in Herráez *et al.*^19^ was applied to recover the phase-unwrapped **Φ**_fw_ and **Ψ**_fw_.

### D. Matrix symmetry and confocal MMF imaging

Numerical matrix compensation has advanced imaging in complex media, with approaches exploiting reflection matrices enabling deep-tissue imaging and spatially distributed aberration correction.^18,20–22^ These frameworks have been extended to accommodate incoherent fluorescence and dynamic scattering.^23,24^ In such methods, the computationally recovered aberrations are inherently constrained by the reciprocity of the overall optical system when imaging in reflection. Our focus in this section is to demonstrate how the restoration of fundamental physical symmetries may complement and enhance the performance of such downstream numerical compensation techniques while imaging in complex media.

**FIG. 3.**
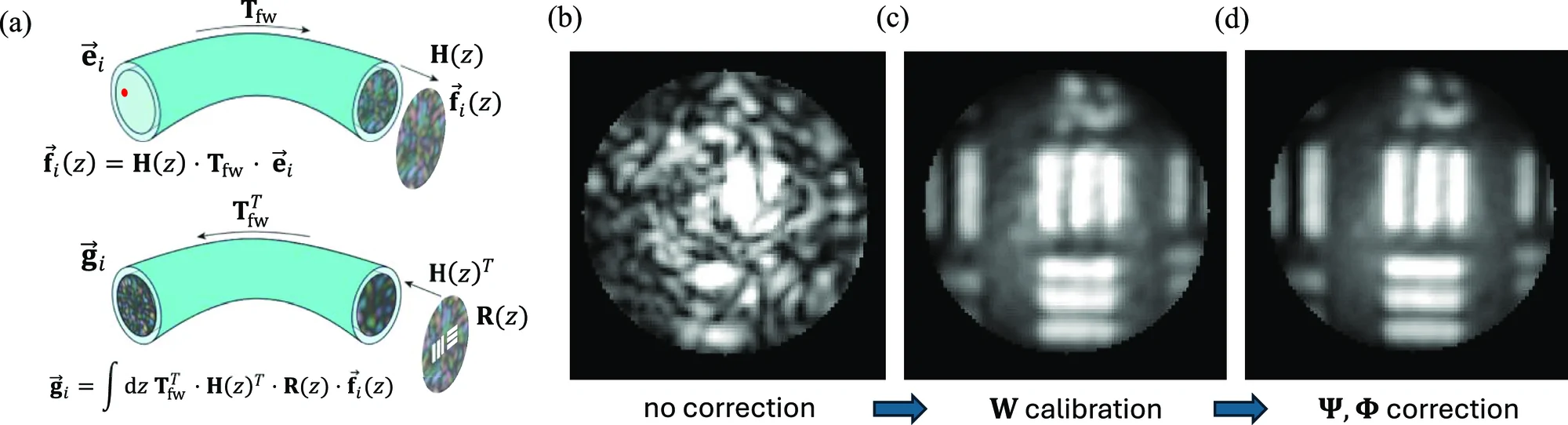
(a) Principle of confocal imaging through MMF without wavefront shaping, in which illumination of a given spot results in a reflected field consisting of propagation through the fiber, free space, and interaction with the sample. (b)–(d) show the reconstruction of the confocal image of a USAF target after application of the proposed symmetrization procedure involving input/output basis alignment (**W**) and compensation of phase aberrations (**Ψ**, **Φ**).

We previously developed computational confocal imaging through MMF without physical wavefront shaping.^8^ In our approach, a sample is placed some distance from the distal MMF facet, and the round-trip TM of the combined system is measured. This matrix, denoted **G** and expressed on a spot-to-spot basis, is given by

$$G=\int dz\;\;T_{\text{fw}}^{T}\cdot H{\left(z\right)}^{T}\cdot R\left(z\right)\cdot H\left(z\right)\cdot T_{\text{fw}},$$ (17)

where **H**(*z*) is the free-space propagation kernel to a given axial position *z* at which **R**(*z*) is the sample’s RM. A schematic illustrating the acquisition of a column (corresponding to a single illumination spot) of **G** is shown in Fig. 3(a). The sample’s RM at a given depth can then be reconstructed by applying the Tikhonov-regularized inverses of the fiber and free-space TMs,

$$R\left(z\right)\approx H{\left(z\right)}^{-T}\cdot T_{\text{fw}}^{-T\;\left(tik\right)}\cdot G\cdot T_{\text{fw}}^{-1\;\left(tik\right)}\cdot H{\left(z\right)}^{-1}.$$ (18)

Confocal gating requires precise alignment of the illuminated and detected fields through the same focal spot. It was therefore hypothesized that to synthesize a meaningful confocal image with **R**, the round-trip matrix **G** must first be transpose-symmetric and expressed in identical input and output bases. Conceptually, it corresponds to ensuring reciprocal overlap of the illuminated and detected foci. In this case, the confocal image of the sample appears on the diagonal of the compensated matrix, **R**(*z*).

To experimentally evaluate this hypothesis, the effect of various steps of the proposed symmetrization procedure on the reconstructed confocal image of a USAF target is plotted in Figs. 3(b)–3(d). In Fig. 3(b), the diagonal of the experimentally measured **R** is taken as the confocal image, without alignment of the input and output bases. Owing to geometric discrepancies between illumination and detection, the image appears scrambled and the USAF target is invisible. In Fig. 3(c), the confocal image is synthesized after the illumination and detection bases are matched using the spot-to-pixel transformation matrix (**W**), resulting in a visible but blurred image. Finally, the compensation of phase aberrations using the proposed symmetrization algorithm results in improved contrast in the confocal image [Fig. 3(d)]. The relatively dominant effect of **W** on restoring the image quality as compared to correction by **Ψ** and **Φ** may be attributed to the significant mismatch between the focal spot and camera pixel grids, as compared to the minor asymmetric tilt in the optical system. In addition, while the demonstrated confocal imaging was scalar in nature, the full vectorial symmetrization framework developed in Secs. II A–II C provides a direct basis for applying such corrections to polarization-sensitive imaging modalities.

## III. NORMALITY IN MULTIMODE FIBER TRANSPORT

### A. The Schur decomposition and spectral energy

While reciprocity provides a criterion for ensuring experimental fidelity, it does not, by itself, assess the intrinsic transport physics of the medium under test. To examine this distinction, attention was turned to the mathematical structure of the measured TMs.

Propagation through guided-wave systems like MMF is expected to occur through orthogonal eigenmodes. In matrix analysis, this is captured by normality: a complex matrix $A\in C^{n\times n}$ is said to be normal if it commutes with its adjoint;^25^ that is, **A** · **A**^†^ = **A**^†^ · **A**. By the spectral theorem, normal matrices admit diagonalization by a unitary operator,^25^ **P**,

$$A=P\cdot \Lambda \cdot P^{†},$$ (19)

where **P** · **P**^†^ = **P**^†^ · **P** = **I**_*n*_ and **P** has orthonormal columns corresponding to the eigenvectors of **A**; **Λ** is a diagonal matrix that contains the associated eigenvalues.^25^ A normal matrix **A** can be interpreted as operating independently on each orthogonal eigenvector. In contrast, non-normal matrices possess non-orthogonal eigenvectors that enable strong modal coupling and transient amplification. Normality thus generalizes unitarity, which imposes the much stricter additional condition that these eigenvalues must be of unit magnitude, implying perfect energy conservation. Physically, this distinction corresponds to energy transfer through orthogonal channels that may be lossy yet remain decoupled.

To assess normality, a modification of Henrici’s departure was used,^26^

$$\eta \left(A\right)=\frac{\sum_{i}|\lambda_{i}{|}^{2}}{\|A{\|}_{F}^{2}},$$ (20)

where $A\in C^{n\times n}$ is a diagonalizable *n* × *n* complex matrix whose eigenvalues are *λ*_*i*_.

**FIG. 4.**
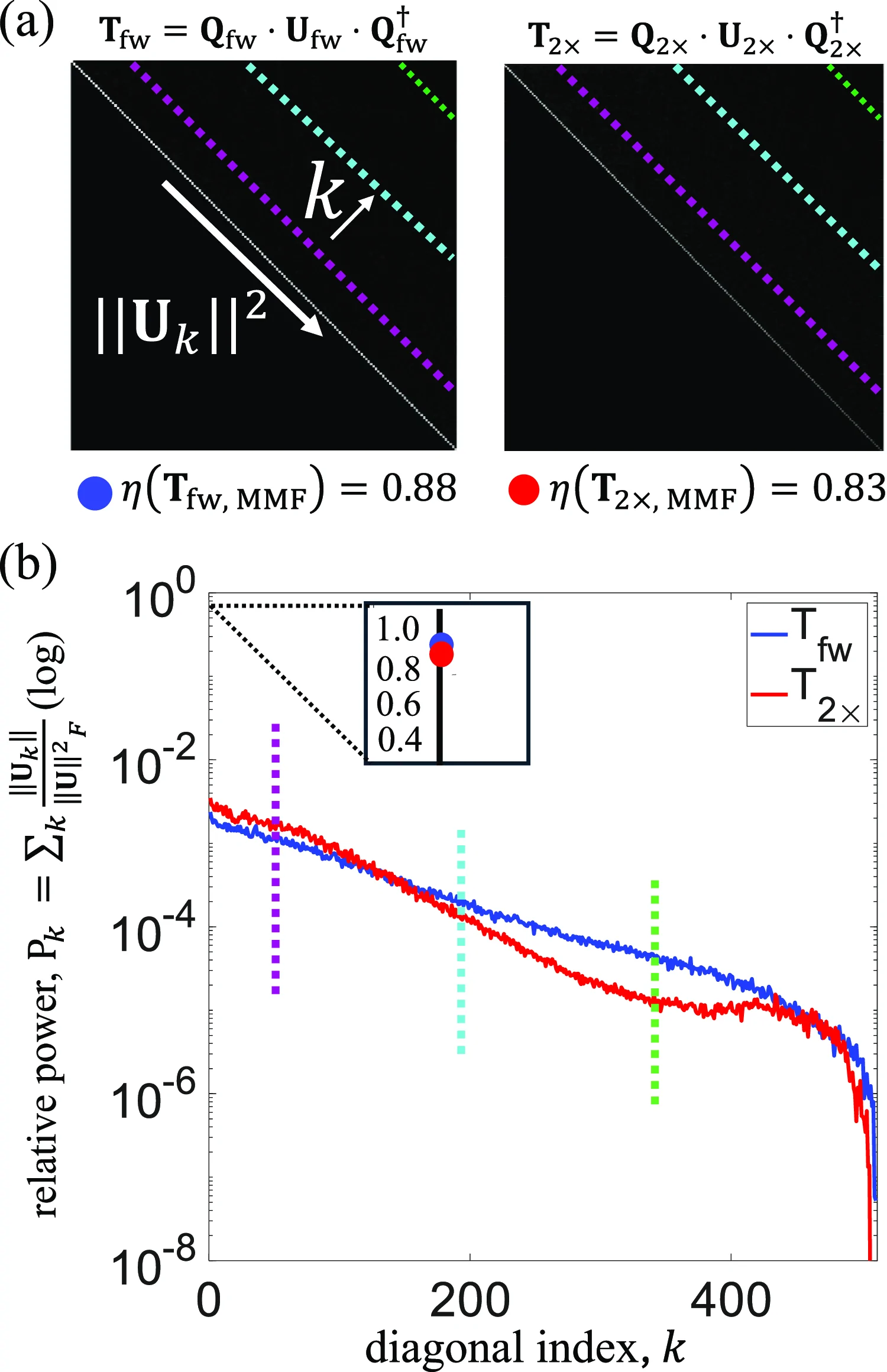
(a) The upper-triangular matrices (**U**_fw_ and **U**_2×_) from the Schur decomposition of the MMF TMs. The diagonal dominance, characteristic of near-normality, is visually apparent, and the calculated normality metric *η* is shown for each. (b) A quantitative comparison of the off-diagonal energy decay. The plot shows the relative power *P*_*k*_ (the sum of squared elements on the *k*th off-diagonal, normalized by the matrix’s total energy versus the diagonal index *k*).

This metric is readily interpreted using the Schur decomposition, which states that any complex matrix **A** may be factored as

$$A=Q\cdot U\cdot Q^{†},$$ (21)

where **Q** is unitary and **U** is an upper-triangular matrix whose diagonal contains *λ*_*i*_. For normal matrices, the Schur and eigenvalue decompositions are equivalent; **P** ≡ **Q** and **Λ** ≡ **U**. Since the Frobenius norm is preserved by the unitary similarity transform^25^ $\left(\|A{\|}_{F}^{2}=\|U{\|}_{F}^{2}\right)$ and the diagonal entries of **U** are the eigenvalues *λ*_*i*_, the normality metric *η*(**A**) = 1 for a normal matrix. More generally, *η* quantifies the fraction of the total operator energy contained on the diagonal of **U** (i.e., by the eigenvalues). A departure from normality (*η* < 1) is contained in the residual energy in the off-diagonal elements of **U**. These terms represent the leakage of energy between the orthonormal columns of **Q**. Figure 4(a) shows the Schur decomposition of the fiber TMs, exhibiting strong diagonality. Calculating the energy in the sub-diagonals [Fig. 4(b)] reveals a sharp decay, characteristic of close-to-normal matrices in which the eigenvalues carry most of the system’s energy.

The normality of the experimentally measured TMs was evaluated using *η*. It was found, however, that a physically meaningful evaluation is highly sensitive to the chosen measurement basis. A physical operator that is intrinsically normal (i.e., possesses orthogonal eigenchannels) may appear non-normal if it is projected onto asymmetric or misaligned illumination and detection bases. Such a basis mismatch itself introduces artificial modal crosstalk, which is indistinguishable from the medium’s true transport behavior. The basis registration procedure, corresponding to right-multiplication by the matrix **W** (as described in Sec. II A), was therefore a prerequisite for a physically meaningful analysis. Its importance was confirmed experimentally. For the unregistered forward MMF TM, the normality was *η*(**T**_fw_) = 57.2%; after registration, this value increased substantially to *η*(**T**_fw_) = 88.2%. A similar increase from *η*(**T**_2×_) = 59.3% to *η*(**T**_2×_) = 82.8% was observed for the double-pass matrix. The decrease in *η* for **T**_2×_ compared to **T**_fw_ was an expected consequence of a longer optical path-length resulting in increased modal crosstalk. Following explicit basis registration, the subsequent application of the aberration correction operators (**Ψ**, **Φ**) yielded only marginal improvements to the matrix normality [e.g., a <1% increase in *η*(**T**_2×_)]. This indicates that the residual, slowly varying phase tilts in the experimental system are insufficient to totally destroy intrinsic modal orthogonality once the bases were roughly aligned.

### B. Normality and principal mode bandwidth

Having established matrix normality as a structural descriptor, its functional implications for characterizing optical media were next examined. As established by Henrici^26^ and others,^27^ non-normal matrices are defined by their spectral fragility; their non-orthogonal eigenbases are highly sensitive to perturbation. In the context of wave systems, a change in optical frequency, denoted Δ*ω*, acts as a natural perturbation on a system’s TM. This led to the hypothesis that the degree of system normality must govern the stability of the system under a perturbative change in optical frequency.

This link between structural stability and frequency perturbation directly connects to the central idea behind the principal mode (PM) basis, computed as the eigenvector matrix of the Wigner–Smith operator^28,29^ $D=-jS^{-1}\frac{\partial S}{\partial \omega }$. The PMs are, by definition, resistant to frequency change to leading order and, in an ideal MMF, correspond exactly to the orthonormal spatial eigenmode basis.^30^ Previous investigations have extensively characterized PMs in MMF, owing to their practical relevance in spectroscopy and telecommunications.^3,13,31–34^

Based on this concept, our group previously developed a parametric approach to model dispersion in MMF.^13^ In that study, a model of the form

$$T\left(\omega_{0}+\Delta \omega \right)=\exp\left(\Omega \left(\Delta \omega \right)\right)\cdot T\left(\omega_{0}\right),$$ (22)

was constructed, where **T**(*ω*) is the MMF TM at a given frequency *ω*, Δ*ω* is a frequency offset from a center frequency *ω*_0_, and **Ω**(Δ*ω*) is modeled as a series expansion

$$\Omega \left(\Delta \omega \right)=\overset{\infty }{\underset{k=1}{\sum }}\Omega_{k}\cdot \Delta \omega^{k}.$$ (23)

The matrix **Ω**_1_ corresponds to the first-order dispersion correction, whose spectral bandwidth directly corresponds to the fidelity of the principal mode basis in characterizing the evolution of **T**(*ω*).^13^ Higher-order correction terms in the expansion are then necessary to account for non-commutative effects, such as modal crosstalk or shifts in the eigenbasis.

To test the hypothesis that the fidelity of a model parameterized only by **Ω**_1_ relies on normality in a medium with fixed chromatic dispersion, multi-spectral MMF TMs (msTMs) were measured under a set of controlled conditions. The experiment began with an unperturbed fiber and proceeded through several stages of increasing static mechanical compression applied using a mode scrambler. Increasing stress was expected to enhance modal crosstalk and induce shifts in the eigenbasis, thereby degrading the accuracy of a first-order dispersion model.

To establish statistical confidence in our ability to differentiate these induced states, we analyzed the variance of the normality metric *η* across the ensemble of measured wavelengths for each condition. For each compression level, a msTM consisting of 40 discrete frequency points was acquired. The normality metric *η* exhibited minimal spectral variance (<1%) within each physical state. Pairwise Welch’s t-tests were then applied to these spectral ensembles to compare the means between compression levels. All comparisons yielded highly statistically significant differences (e.g., *p* ≪ 0.01 within each group of 40 TMs). This justified using its value at the center wavelength for all subsequent analyses without loss of generality.

**FIG. 5.**
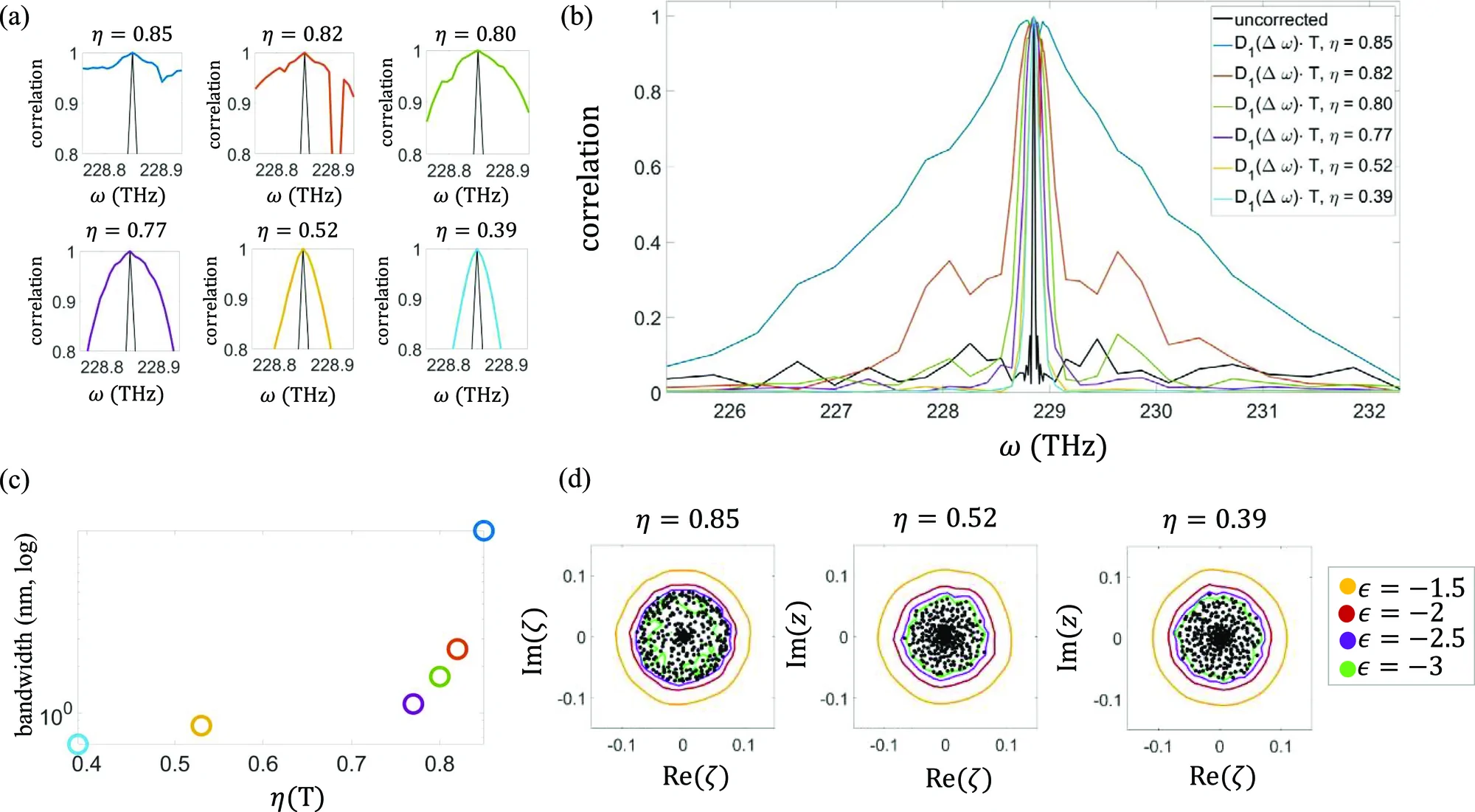
(a) First order msTM dispersion model over a 1 nm bandwidth centered around 1310 nm for varying levels of normality. The black curves show the spectral decorrelation of the uncorrected TM with wavelength. The colored curves show the performance of a first-order dispersion model applied to TMs of progressively lower normality, from nearly normal (*η* = 0.85, blue curve) to highly non-normal (*η* = 0.39, light blue curve), due to mechanical compression applied by a fiber-optic mode scrambler. (b) Application of the first-order dispersion model over a 40 nm wavelength range. (c) The extracted approximate 0.50 correlation bandwidth (nm) plotted as a function of the TM’s normality, *η*. (d) The nearly normal, unperturbed MMF (*η* = 0.85) exhibits tight, circular *ɛ*-pseudospectral contours (colored lines, *ɛ* represented in log_10_ scale) around its eigenvalues (black dots), characteristic of a spectrally stable operator. In contrast, the mode-scrambled, low-normality TMs (*η* = 0.52 and *η* = 0.39) display progressively “bloated” pseudospectra, revealing the spectral instability responsible for the collapse of the predictive dispersion model shown in (a)–(c).

The first order dispersion model was constructed according to the procedure established by Lee *et al.*,^13^ using a msTM of *N*_narrow_ = 21 TMs spaced by a constant frequency difference of Δ*ω* = 8 GHz surrounding *λ*_0_ = 1310 nm. The fidelity of the constructed first order model over the measured narrow wavelength range is shown in Fig. 5(a). To evaluate its validity over a broader frequency range, a subsequent msTM of *N*_broad_ = 30 TMs was sampled between wavelengths of 1290 and 1330 nm. The fidelity of the linear dispersion model, evaluated across wavelengths and perturbative conditions, is shown in Fig. 5(b).

To directly quantify the link between normality and model fidelity, the 0.50 cosine similarity bandwidth was extracted for each level of mechanical compression and plotted as a function of the corresponding TM normality, *η* [Fig. 5(c)]. A monotonically increasing relationship was observed. As the induced mechanical stress reduced the operator’s normality, the predictive bandwidth of the first-order model systematically collapsed, reducing from ∼2.60 THz in the nearly normal case (*η* = 0.85) to 0.11 THz in the highly non-normal regime (*η* = 0.39).

The spectral stability of the measured matrices is directly visualized using their *ɛ*-pseudospectra, shown in Fig. 5(d). While a system’s eigenvalues describe its ideal response to an eigenvector, the pseudospectrum reveals how sensitive that response is to small perturbations.^27^ For a spectrally stable (nearly normal) system, the pseudospectral contours are tight circles around the eigenvalues, meaning small perturbations cause only small changes in the system’s response to an eigenvector. For an unstable (non-normal) system, these contours become large and bloated, indicating that a small perturbation can trigger a dramatic shift in the system’s behavior.^27^ As a result, in non-normal media, the eigenstructure is of little use in characterizing the system, and its eigenmodes have no well-defined meaning. The *ɛ*-pseudospectrum formalizes this for a perturbation of a given magnitude *ɛ* (logarithmic scale, shown as colored lines) by mapping the potential locations of eigenvalues (shown as black dots).

By mapping the perturbative response of each eigenvalue in turn, *ɛ*-pseudospectrum comprehensively maps the structural fragility across all spatial channels, revealing that in highly non-normal systems, certain eigenchannels may exhibit extreme sensitivity to perturbation while others remain robust. In the present investigation, the induced mechanical compression admittedly resulted in a relatively uniform expansion of the pseudospectral contours across all eigenvalues [Fig. 5(d)], which did not fully exercise the channel-specific diagnostic power of the pseudospectrum. However, it may prove useful in analyses of fragilities in systems subject to more localized or structured responses to perturbation.

## IV. DISCUSSION

Reciprocity and matrix normality impose independent yet complementary characterizations on the mathematical structure of MMF transport. Reciprocity arises from the fundamental symmetry of the wave equation under source-observer interchange and applies universally to all linear, source-free systems. Matrix normality, by contrast, reflects the intrinsic orthogonality of the propagating eigenmodes within a specific optical system.

While our symmetrization procedure effectively compensates for experimental asymmetries, it is important to define its operational bounds. Fundamentally, the algorithm makes no assumptions regarding the internal propagation physics, geometry, or refractive index profile of the medium under test; in addition, it operates in a reciprocal regime. The method thus generalizes well to longer fibers, higher mode counts, and graded-index profiles; indeed, our experiments confirmed its applicability to purely scattering media (supplementary material File 1). Furthermore, the extension to vectorial fields in the present study enabled use of the distal facet as a reflector, which was previously not possible with scalar waves due to polarization crosstalk within the fiber’s TM.^17^

However, the linear nature of the applied correction terms means that nonlinear asymmetries cannot be corrected by the optimization procedure. In our experiments, for example, it could not resolve the initial misalignment between the illumination and detection bases because the dominant mismatch was a geometric scaling (demagnification) between the focal spot and camera pixel grids. Consequently, the explicit **W** matrix calibration was a strict prerequisite to the optimization procedure. We also assumed, based on the physical construction of our experimental setup, that the dominant source of polarization effects was the fiber-based illumination path. Because we chose to correct for output-side effects, these input polarization state effects commute with our choice of a polarization-preserving far field term **Φ** and result in the block-Jones format of the near field term **Ψ**; however, a more comprehensive formulation could have included polarization-dependent behavior also in the far-field term. Moreover, by constraining the correction operators to diagonal matrices, the method implicitly assumes that the system-level aberrations are spatially local and non-mixing (i.e., occurring over an isoplanatic patch). As a result, this approach can correct phase aberrations such as defocus or tilt, but it cannot compensate for volumetric scattering within the experimental apparatus itself (e.g., effects that require a full, non-diagonal TM to describe).

Just as explicit input–output registration was necessary to observe reciprocity, it was equally essential for establishing matrix normality. Notably, *η* did not depend on the specific choice of basis, but rather on the strict geometric alignment between input and output sampling. Expressing **T**_2×_ and **T**_fw_ in both pixel-to-pixel and spot-to-spot bases—the latter constructed by convolving the average point spread function of the input focus across the output camera image—yielded similar values of *η*. This interpretation of normality as a physically meaningful, basis-aligned property offers insight into previous results. For instance, the aberration correction demonstrated by Matthès *et al.* to deduce the mode-basis MMF TM^12^ may be viewed through the lens of normality optimization. By applying Zernike-based phase corrections to maximize energy in a predefined mode basis, they observed the MMF TM becoming approximately diagonal, concentrating 92% of the energy in the mode basis. This is in rough agreement with the normality of the TMs examined here, though our investigation utilizes the Schur decomposition to quantify normality intrinsically, without requiring prior knowledge of the underlying modal basis.

Previous studies have also established the link between mode-coupling and PM behavior, demonstrating how spatial and polarization crosstalk in MMF induces collapse of PM bandwidth.^3,33–35^ Our exploration of the relationship between matrix normality and PMs offers a complementary lens of understanding these phenomena. Because PMs are found via a numerically computed derivative through the Wigner–Smith operator, they are not obviously related to the medium’s spatial eigenmodes in practice. This disparity has decoupled experimental analyses of eigenmodes from those of PMs, though theory dictates that they are the same in an ideal MMF.^30^

In contrast, matrix normality serves as a static, empirical descriptor that directly evaluates the orthogonality of the measured operator’s eigenmodes from a monochromatic measurement without explicit specification of the parameter of perturbation or differentiation of the scattering operator. The collapse of PM bandwidth accompanied by a decrease in normality thus demonstrates how a broadband waveguide property can be inferred directly by inspecting spatial behavior, unifying the relationship between spatial and spectral properties of MMF. In addition, matrix normality can be applied to usefully characterize systems in which a theoretical mode basis (e.g., LP or HG modes) does not exist or is unknown. Because it relies on the empirical eigenmodes derived directly from the measured operator, normality provides a model-agnostic metric of mode orthogonality that does not force the system to fit a preconceived and idealized waveguide geometry. This may enable normality to be compatible with analysis of more general scattering media and optical systems beyond waveguides or fibers.

## V. CONCLUSION

In this study, a compensation procedure to restore reciprocity-imposed symmetries to polarization-resolved TMs of MMF was developed. The illumination and detection bases were first explicitly co-registered using a direct transformation matrix. An optimization procedure was then developed that uses the underlying symmetry constraints in the objective function to recover the compensatory aberrations without parameterization. The restoration of these symmetries was found to be critical to our previously developed approach for computational confocal imaging through MMF.

Using these matrices, matrix normality was explored as a quantitative metric of modal orthogonality in MMF systems. By introducing increasing levels of mechanical compression to the fiber, we observed a monotonic relationship between normality and the spectral correlation bandwidth of the MMF’s principal mode basis. Normality thus affords a simple way to assess the spectral characteristics of the fiber solely from a monochromatic measurement.

## SUPPLEMENTARY MATERIAL

See the supplementary material for additional experimental results regarding the restoration of reciprocity-imposed symmetries in scattering media and further discussion of the symmetry-enforcement algorithm.

## Figures and Tables

**FIG. 6.**
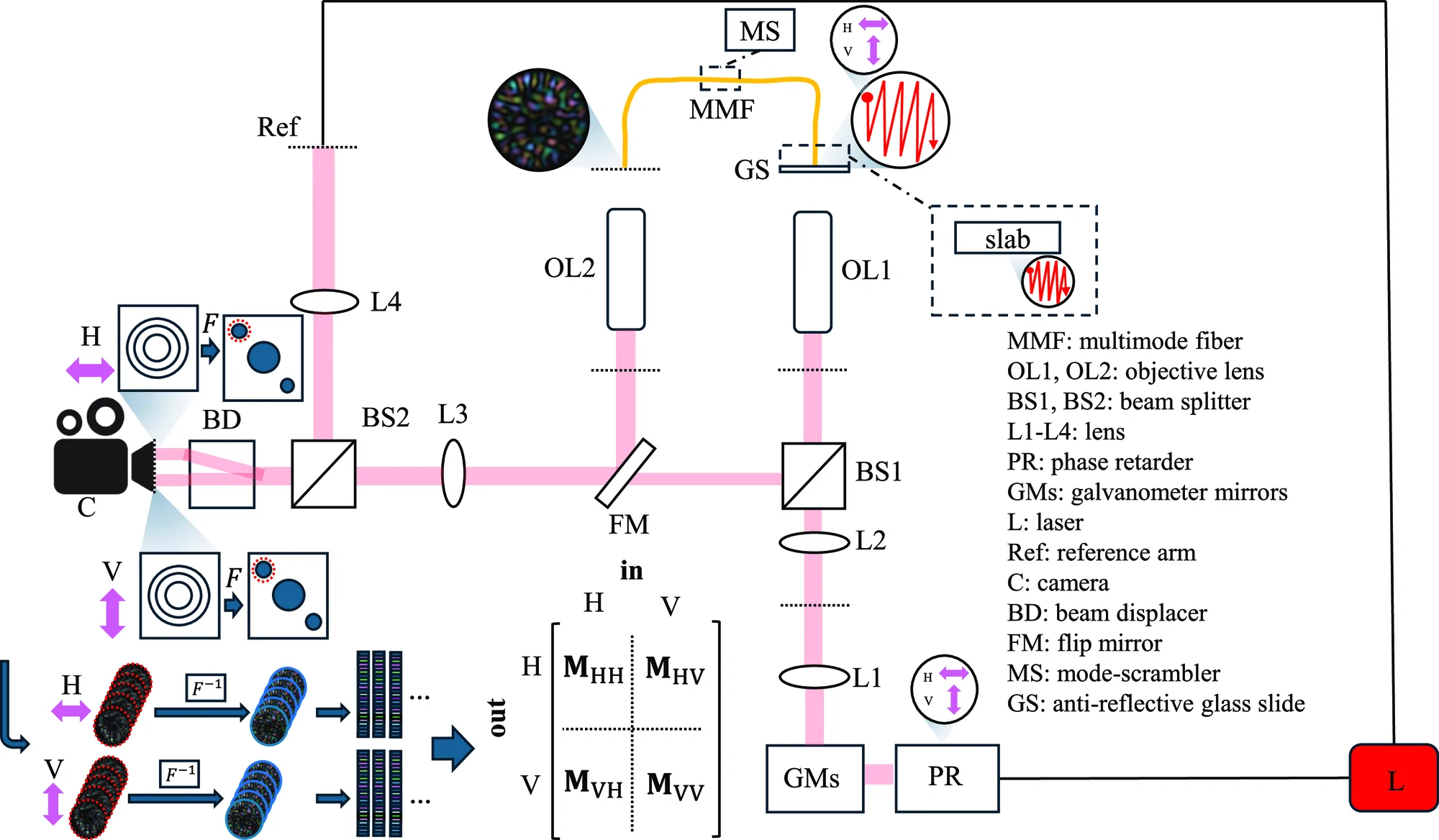
Experimental setup and procedure used in the present investigation to measure MMF transmission matrices. The proximal facet was scanned with a grid of focal spots, and the output complex field was measured using off-axis holography.

## Data Availability

The data that support the findings of this study are available from the corresponding author upon reasonable request.

## APPENDIX A: EXPERIMENTAL SETUP

The experimental system is shown in Fig. 6. A wavelength-tunable semiconductor laser (L, TSL-570, Santec) with a <100 kHz linewidth and tuning range of 1260–1360 nm was used as the source. The source was split into sample and reference arms in a Mach–Zehnder interferometer configuration. A two-dimensional galvanometric mirror scanner (GMs, GVSM002, Thorlabs) was used to sequentially scan the focal spot across a lateral grid to define a 16 × 16 grid of input spatial channels. For each spot, the polarization state was switched between orthogonal horizontal (H) and vertical (V) linear states using a fiber-coupled electro-optic phase retarder (PR, PRT1010, Boston Applied Technologies Inc). The proximal facet of the MMF was kept at the focal plane of the first objective lens (OL1, NIR Infinity Corrected, Mitutoyo, NA 0.4, f = 10 mm, 20×). For forward MMF TM measurements, the distal facet was placed at the focal plane of a second objective lens (OL2, NIR Infinity Corrected, Mitutoyo, NA 0.4, f = 10 mm, 20×). The distal facet of the fiber was imaged, together with the reference arm, added through a beam splitter (BS2) onto the camera after being split into orthogonal polarization components using a beam displacer (BD, BD40, Thorlabs). For round-trip MMF TM or RM measurements, the flip mirror was toggled such that light was redirected to be imaged onto the camera by OL1 through a beam-splitter in the illumination path (BS1).

To investigate regimes with increased mode coupling, a fiber-optic mode scrambler (FM-1, Newport Corporation) was selectively used to introduce controlled mechanical compression to the MMF. Detection was performed with an InGaAs camera (OW1.7-VS-CL-LP-640, Raptor Photonics), with a 20 *µ*s exposure time and a maximum frame rate of 120 Hz. Off-axis digital holography was employed to recover the complex-valued field using computational demodulation in the spatial frequency domain.^36^ A fixed reference input spot was revisited after each input mode during TM acquisition to enable phase tracking and compensating for phase drift. The recorded interferograms were demodulated by correcting for the spectral variation of the off-axis carrier and aligned using the phase-tracking data. The complex fields were flattened into column vectors and assembled into a TM or RM, represented as a block-Jones matrix. The MMF experiments were performed using a 0.22 NA, 50 *μ*m diameter, step-index MMF (FG050LGA, Thorlabs, supporting 170 modes per polarization).

## APPENDIX B: REGISTRATION OF ILLUMINATION AND DETECTION BASES

Our experimentally measured matrices (**M**) were initially acquired using a 16 × 16 grid of input spots per polarization and an output basis defined by a 109 × 109 array of camera pixels. Since each column of (**M**) was the vectorized camera image of the transmitted or reflected fields, raw matrices were of size 2 × 109^2^ × 2 × 16^2^, where the factor of two accounts for polarization multiplicity. These matrices were non-square and, therefore, unsuitable for interrogation of their mathematical properties. To obtain a square, physically interpretable form, the demodulated off-axis holograms, originally recorded on the full 109 × 109 grid of camera pixels, were cropped to a 16 × 16 grid in the spatial frequency domain, inverse Fourier-transformed, and vectorized to yield a 2 × 16^2^ × 2 × 16^2^ matrix. This Fourier-domain subsampling procedure produced symmetric dimensionality between input and output.

This resizing, however, did not align the distinct input (focal spot) and output (camera pixel) bases. To register them, a spot-to-pixel transformation operator, denoted **W**′, was constructed from a direct calibration measurement. The fiber was removed from the experimental apparatus, leaving a glass slide used to minimize Fresnel reflection at the air-fiber facet interface. The reflected focal spot array was imaged directly onto the camera through the illumination objective. The centroid of each spot was identified, and the mean point-spread function (PSF) of the spots was fit to a truncated Gaussian profile. By placing this PSF at each of the measured spot centroids on the pixel grid, a set of 16^2^ images, each corresponding to a spatially separated, i.e., orthogonal input mode, was constructed. Each image, originally of dimension 109 × 109, was then Fourier-domain–subsampled to the 16 × 16 grid.

Each column of **W**′ was formed by vectorizing the resulting subsampled pixel distribution of an individual input spot. **W**′ thus represents the projection of the input spot basis onto the output subsampled-pixel basis. Applied independently to both polarization channels, it defines the block-diagonal operator

$$W=\left[\begin{array}{ccc} W^{\prime } & & 0\\ 0 & & W^{\prime } \end{array}\right],$$ (B1)

which converts from the spot basis to the subsampled-pixel basis. Since the columns of **W**′ (and, therefore, **W**) are spatially orthonormal input spots, **W**^−1^ = **W**^†^, and right multiplication of the output-subsampled **T**_2×_ by **W**^†^ converts the input representation from the focal-spot basis to the subsampled-pixel basis. This transformation resulted in square matrices with matching input and output bases and was applied to all TMs prior to further analysis. For the forward MMF TM, the validity of this registration procedure relied on the careful experimental co-registration of the proximal facet (imaged during the **T**_2×_ measurement) and the distal facet (imaged during the **T**_fw_ measurement) onto the same camera pixel grid.
